# The acceptability and feasibility of a brief psychosocial intervention to reduce blood-borne virus risk behaviours among people who inject drugs: a randomised control feasibility trial of a psychosocial intervention (the PROTECT study) versus treatment as usual

**DOI:** 10.1186/s12954-017-0142-5

**Published:** 2017-03-21

**Authors:** Gail Gilchrist, Davina Swan, April Shaw, Ada Keding, Sarah Towers, Noel Craine, Alison Munro, Elizabeth Hughes, Steve Parrott, John Strang, Avril Taylor, Judith Watson

**Affiliations:** 1 Institute of Psychiatry, Psychology and Neuroscience, King’s College London, Addictions Sciences Building, 4 Windsor Walk, Denmark Hill, London, SE5 8BB England, UK; 2 School of Media, Culture & Society, University of the West of Scotland, Paisley Campus, High Street, Paisley, PA1 2BE Scotland, UK; 3 York Trials Unit, Department of Health Sciences, University of York, Heslington, York, YO10 5DD England, UK; 4 Betsi Cadwaladr University Hospital Trust, 10 Grove Road, Wrexham, LL11 1DY Wales, UK; 5 Public Health Wales, Microbiology Department, Ysbyty Gwynedd, Bangor, Gwynedd LL57 2PW Wales, UK; 6 School of Health and Human Sciences, University of Huddersfield, Queensgate, Huddersfield, West Yorkshire HD1 3DH England, UK; 7 Department of Health Sciences, University of York, Heslington, York, YO10 5DD England, UK

**Keywords:** Blood-borne virus transmission, People who inject drugs, Feasibility randomised controlled trial, Psychosocial interventions, Focus group research

## Abstract

**Background:**

While opiate substitution therapy and injecting equipment provision (IEP) have reduced blood-borne viruses (BBV) among people who inject drugs (PWID), some PWID continue to share injecting equipment and acquire BBV. Psychosocial interventions that address risk behaviours could reduce BBV transmission among PWID.

**Methods:**

A pragmatic, two-armed randomised controlled, open feasibility study of PWID attending drug treatment or IEP in four UK regions. Ninety-nine PWID were randomly allocated to receive a three-session manualised psychosocial group intervention and BBV transmission information booklet plus treatment as usual (TAU) (*n* = 52) or information booklet plus TAU (*n* = 47). The intervention was developed from evidence-based literature, qualitative interviews with PWID, key stakeholder consultations, and expert opinion. Recruitment rates, retention in treatment, follow-up completion rates and health economic data completion measured feasibility.

**Results:**

Fifty-six percent (99/176) of eligible PWID were recruited. More participants attended at least one intervention session in London (10/16; 63%) and North Wales (7/13; 54%) than in Glasgow (3/12; 25%) and York (0/11). Participants who attended no sessions (*n* = 32) compared to those attending at least one (*n* = 20) session were more likely to be homeless (56 vs 25%, *p* = 0.044), injected drugs for a greater number of days (median 25 vs 6.5, *p* = 0.019) and used a greater number of needles from an IEP in the last month (median 31 vs 20, *p* = 0.056). No adverse events were reported. 45.5% (45/99) were followed up 1 month post-intervention. Feedback forms confirmed that the intervention was acceptable to both intervention facilitators and participants who attended it. Follow-up attendance was associated with fewer days of injecting in the last month (median 14 vs 27, *p* = 0.030) and fewer injections of cocaine (13 vs 30%, *p* = 0.063). Analysis of the questionnaires identified several service use questionnaire categories that could be excluded from the assessment battery in a full-randomised controlled trial.

**Conclusions:**

Findings should be interpreted with caution due to small sample sizes. A future definitive RCT of the psychosocial intervention is not feasible. The complex needs of some PWID may have limited their engagement in the intervention. More flexible delivery methods may have greater reach.

**Trial registration:**

ISRCTN66453696

**Electronic supplementary material:**

The online version of this article (doi:10.1186/s12954-017-0142-5) contains supplementary material, which is available to authorized users.

## Background

Studies report the prevalence of Hepatitis C virus (HCV) among people who inject drugs (PWID) ranges from 5 to 90% [1] and the prevalence of HIV ranges from <1 to 50% [2]. In the UK, HCV is the most prevalent blood-borne virus (BBV) among PWID, with 23–61% being HCV positive [1, 3]; the rates of human immunodeficiency virus (HIV) and hepatitis B (HBV) among PWID in the UK are much lower, 0–1.4% for HIV and 6–18% for HBV [3] preventing the transmission of BBV among PWID thus remains a major public health issue.

While HBV and HIV are transmitted via blood or body fluids, the greatest risk of HCV transmission among PWID is via blood from sharing needles and other injection paraphernalia [4, 5]. Advances have been made in treatment and pre-exposure prophylaxis for HIV [6, 7], and a vaccine is available for HBV [8]; however, there is currently no vaccine available to prevent HCV infection. Opiate substitution therapy and injecting equipment provision (IEP) have been shown to be effective in reducing HIV and HCV among PWID [9–12]; and psychosocial interventions (such as brief interventions, motivational interviewing, cognitive behavioural therapy and contingency management) could further decrease BBVs [10] by educating PWID about transmission risks and developing strategies to avoid them.

Research suggests there is a gap in knowledge among PWID regarding HCV transmission which is contributing to the high prevalence [13–15], among both new and longer term injectors. PWID with mental health issues report greater sharing of injection equipment, lower rates of condom use, multiple sexual partners, sex trading and having sex with PWID [16–19].

Public Health England’s *Shooting Up* report [20] highlighted that in 2015 in England, Wales and Northern Ireland, sharing of needles in the previous month was reported by 16% of individuals attending drug treatment services, in Scotland this figure was 15% in 2014–2015. The report highlighted that the sharing of mixing containers and filters was almost twice as common as the sharing of needles and syringes. A large UK survey has also identified an increased risk of infection for those who inject amphetamines and amphetamine-type drugs, such as mephedrone [21]. Therefore, reducing BBV transmission risk behaviours among PWID remains a priority.

A recent meta-analysis found that interventions using strategies that combined substance-use treatment and support for safe injection were most effective at reducing HCV seroconversion [22]. A number of recent systematic reviews of psychosocial interventions (e.g. skills training, peer-education training and counselling) compared to lesser interventions or educational interventions to reduce HIV and HCV injecting and sexual risk behaviours among PWID have reported modest effects [23, 24], concluding that future research should determine whether these interventions work better for particular groups of drug users [23] and that “multi-component interventions are required” [24].

A psychosocial intervention (the PROTECT intervention) to reduce BBV transmission risk behaviours and increase BBV transmission knowledge among PWID was developed, and a feasibility randomised controlled trial (RCT) comparing the psychosocial intervention to an information leaflet, to demonstrate the feasibility and acceptability of delivering the intervention in harm reduction settings throughout the UK was conducted.

## Methods

### Study design

A pragmatic, two-armed, randomised controlled, open feasibility study in which a psychosocial group (brief) intervention was compared to treatment as usual (TAU) plus information leaflets, on reducing the BBV transmission risk behaviours for PWID aged ≥18 years. Ethical approval was granted by the National Research Ethics Committee East Midlands-Leicester South Research Ethics Committee (Reference: 15/EM/0413). Local Research and Development (R&D) approval was obtained, as was agreement to participate from the relevant services.

### Setting

The trial was conducted in four locations across the UK: in England (London, York), Wales (North Wales) and Scotland (Glasgow). A mix of urban and semi-rural community services/sites were included to ensure different modes of service delivery were represented:

1. *London*: Three Drug and Alcohol Treatment Services providing services including advice and IEP and treatment to people, aged over 18, who have drug- and/or alcohol-related problems, including a prescribing clinic within a hostel for homeless people. The intervention was delivered at one Drug and Alcohol Treatment Service.
2. *York*: Participants were recruited, and the intervention was scheduled to be delivered at a city centre substance use treatment service providing counselling and advice, IEP, condoms, assessment and referral to residential rehabilitation, specialist drug units and other agencies providing treatment for addiction and BBV testing.
3. *Glasgow*: Participants were recruited from and the intervention delivered in a drugs treatment service in the city centre which provides both treatment and IEP.
4. *North Wales*: Participants were recruited from a drug service, a drop-in centre and IEP for homeless people and a mobile harm reduction service for PWID not currently engaged in treatment. The intervention was delivered at the drop-in centre and IEP for homeless people.

### Participants

#### Identification, eligibility and consent

Potential participants were approached by researchers in the waiting rooms of participating services and given a Participant Information Sheet that was also explained to them verbally. Key workers and IEP workers also referred eligible clients to the researchers. In addition, flyers were distributed and posters were displayed in the services, inviting interested participants to contact the researcher for more details about the study. All interested clients were screened for eligibility. Clients were eligible if they were aged ≥18 years, had injected drugs (other than performance enhancing drugs) at least once in the past 4 weeks, planned to stay in the area for the next 3 months, were able to complete the assessments (all assessments were researcher administered) and could communicate in a group intervention in English. They were excluded if they were too intoxicated to give informed consent or were noticeably in withdrawal. If eligible and interested, written informed consent was obtained.

### Outcome Measures

Self-reported age, recent drug use, length of injecting career, drug treatment history, HIV and Hepatitis C status and vaccination against Hepatitis B were recorded.

#### Recruitment and acceptability

Recruitment rates (i.e. number agreeing to participate/number eligible), retention in treatment (number of sessions attended) and follow-up questionnaires completion rates measured feasibility. Acceptability to participants was ascertained through feedback forms and separate focus group discussions with participants and facilitators.

#### Patient-reported outcomes

Participants received £10 cash (London) or £10 gift voucher (York, Glasgow, North Wales) for time involved in completing baseline and follow-up questionnaires. Travel reimbursement was available in London and North Wales. Demographic data were collected at baseline, and the following outcome measures were collected at baseline, at the end of intervention and 1 month post-intervention (intervention arm), and equivalent time period for control arm:

- Injecting risk behaviours and self-efficacy
  Nine injecting risk behaviours were assessed during the past 28 days (including passing any needles or syringes, cleaned needles or syringes, spoons or containers for mixing, or filters to someone else after using them; using any needle or syringes, cleaned needles or syringes, spoons or containers for mixing, or filters previously used by someone else; sharing rinse water) that may have exposed them to BBV in the previous month were assessed using questions from Public Health England’s survey of PWID [3]. Events were summed to a total ranging from 0 (engaged in no risk events) to 9 (engaged in all of the risk events). Participants indicated agreement with eight self-efficacy questions around injecting behaviours, e.g. “I can avoid sharing a needle even if I am in withdrawal”, around injecting skills (including finding a vein, sharing equipment, cleaning equipment and talking about safe drug use) [25]. Agreement was rated between 1 (absolutely cannot) and 4 (absolutely can), with total scores between 8 (low self-efficacy) and 32 (high self-efficacy) [25].
- Sexual risk behaviours
  Having had sex with more than one partner in the past month, not always having used a condom when having sex in the past month, four items assessed whether participants were not “absolutely sure” they would use a condom in a given situation (sex with a regular partner, even if they do not want to use one or participant had been using alcohol or drugs; sex with a casual partner, even if they do not want to use one or participant had been using alcohol or drugs) and one question on whether they would be able to talk about safe sex with sexual partners they did not know. These seven items were summed to a total ranging from 0 (no risk behaviours) to 7 (all risk behaviours).
- Withdrawal Prevention Tactics scale
  This five-item scale asked whether participants had done any of four listed tactics to avoid withdrawal episodes: saved a bag for the next morning, put aside additional drugs, stored methadone or put aside money for getting the next bag in an emergency [26]. A fifth item asked about use of other substances, such as painkillers, to avoid withdrawal symptoms until they are able to obtain their drug of choice. The frequency of undertaking each withdrawal activity in the past month were collected with responses ranging from 0 (never) to 4 (very often). The total score ranged from 0 (never taken any of the preventative actions) to 20 (taken preventative actions very often for all of the activities).
- BBV transmission knowledge
  Participants rated 14 statements about HIV transmission [27], 31 about HCV transmission [28, 29] and 15 about HBV [30]. The total number of correct answers across each BBV transmission questionnaire was summed (range 0–14 for HIV, 0–31 for HCV and 0–15 for HBV).
- Motivation to change behaviour
  Participants were asked to rate their motivation from 1 (not at all motivated) to 5 (extremely motivated) to protect themselves and others from acquiring BBV.
- Health-related quality of life (HRQoL)
  The European quality of life-5 dimensions-5 levels (EQ-5D-5L) characterised health on five dimensions (mobility, self-care, ability to undertake usual activities, pain, anxiety/depression) [31].
- Health and social resource used
  Hospital and primary health care services use, drug service use, other health-related services, contact with the police and criminal justice system, and medications prescribed in the past month were recorded.

#### Sample size

We aimed to recruit a total of 128 participants (64 in intervention group) from harm reduction services in 4 locations (Glasgow, London, York and North Wales), exceeding that recommended for feasibility studies of between 24 and 50 [32–34] and allowed feasibility assessments within both community clinics and IEP.

#### Randomisation process

Treatment allocation was performed by a secure, remote, telephone randomisation service based at the University of York. Participants were randomised by stratified block randomisation, ensuring balanced allocation within each location, setting (community drug service or IEP) and gender. Participants were randomised to either:

- The psychosocial group intervention, information booklets plus TAU or
- The control arm: information booklets plus TAU only

#### Intervention and comparator

The PROTECT intervention was co-developed by service users, service providers, policy makers and academics based on an evidence-based literature, qualitative interviews with PWID, consultation with key stakeholders and expert opinion. The PROTECT manual is available for download free of charge via: https://www.kcl.ac.uk/ioppn/depts/addictions/research/drugs/bloodborneviruses.aspx. The manualised psychosocial group intervention consisted of three, one hour sessions (preferably, one a week for three consecutive weeks). Session 1 covered improving injection skills and good vein care. Session 2 covered planning for risk situations. Session 3 provided information about blood-borne viruses and transmission risk behaviours. Sessions used videos, games and exercises to facilitate discussion and build skills and strategies to reduce and avoid risk. All sessions also included a didactic education section. Separate groups were held for women and men.

The structure of drug treatment services was different across the settings in the study; the precise job role of the health care professionals who conducted the groups thus varied. However, in all settings, groups were facilitated by professionals with considerable experience of working with PWID and BBV. Training took place with all facilitators in London over 1 day and was co-delivered by a clinician and peer educator. Following training, intervention delivery varied across sites to reflect current service provision: London—the group was co-facilitated by a drug worker and peer educator (gender of co-facilitators matched that of the gender of the group); Glasgow—groups were co-facilitated by one male drug worker and one female project co-ordinator; North Wales—the groups were co-facilitated by one male and one female drug worker; and York—the groups were due to be delivered by one male nurse specialising in BBV prevention and treatment. Contingency management was used to retain participants in the intervention [35]. Participants allocated to the intervention arm received £10 cash (London) or £10 gift voucher (Glasgow and North Wales) for each of the three sessions attended. A “bonus” of £10 cash (London) or £10 gift voucher (Glasgow and North Wales) was given to those who attended all three sessions.

##### Intervention evaluation/fidelity

All sessions were observed by at least one researcher to assess the feasibility of the quality assurance methods proposed for the main trial, including acceptability to drug worker/nursing staff and service users. A brief checklist was used to identify what aspects of the intervention manual were implemented. At the end of each session, facilitators and participants rated the session using an evaluation form developed for the study.

### Control

Participants in both arms received TAU from the service from which they were recruited and a booklet containing information on Hepatitis C (*“Hep C Info: Understanding hepatitis C and staying safe”* http://ljwg.org.uk/ljwg-toolkit/resources/) and a one-page information sheet developed specifically for the trial about a recent HIV outbreak among PWID.

### Analyses

**Fig. 1:**
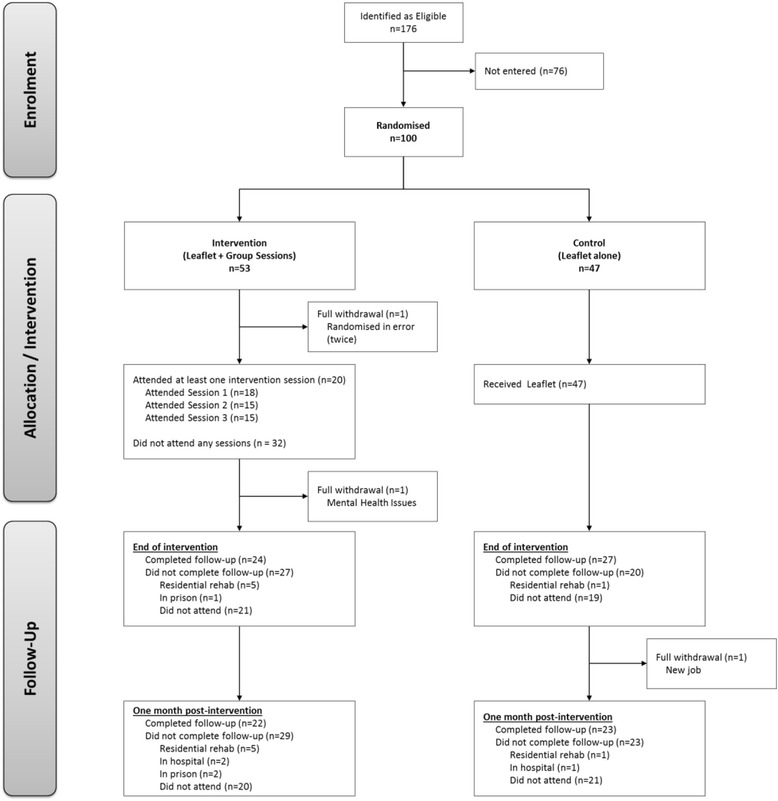
Study flow diagram

All analyses were conducted using Stata Version 13.1. Feasibility parameters were reported descriptively and participant flow is illustrated with a flow diagram [Fig. 1: Study flow diagram]. Following observed differences in compliance and follow-up at the four sites, population characteristics for these groups were compared using Fisher’s Exact and Wilcoxon rank-sum tests. As a feasibility trial, the study was not powered for formal testing of intervention effectiveness; however, group differences for selected outcome measures were explored as follows. Longitudinal regression analyses for each outcome at the two follow-up points were conducted, adjusting for baseline values, gender and recruitment site. Estimated mean treatment group differences from these analyses are presented by intention to treat (ITT) and per protocol groups together with 80 and 95% confidence intervals.

The economic analysis included intervention costing, calculation of NHS and wider social costs per patient, EQ-5D-5 L results and assessment of the pilot questionnaires. Quantities of service use recorded were multiplied by national average unit costs of health care and criminal justice contacts to derive a health care cost (price year 2014/5). Follow-up costs were defined by summing costs at the end of the intervention and one month post-intervention.

## Results

### Feasibility parameters

Feasibility was assessed as the proportion of patients consented and randomised, as well as compliance with the intervention and attrition throughout follow-up.

The flow of participants is shown in Fig. 1 [Fig. 1: Study flow diagram]. Of 176 eligible people who injected drugs, 99 (56%) individuals were randomised into the feasibility trial during January and February 2016. One person was mistakenly randomised twice (therefore, 100 randomisations); their second randomisation was subsequently withdrawn.

It was not possible to compare those who were eligible that did and did not agree to participate. The eligibility question asked whether potential participants had injected drugs in the previous month. If they had, researchers discussed the study with potential participants and what taking part involved. Seventy-seven of the 176 eligible participants (44%) did not take part as they were not interested, too busy, entering rehabilitation treatment, too ill to participate, unavailable to attend interventions, not wishing others to know about their injecting, declined without reason, or were uncontactable or did not attend their baseline appointment.

Fifty-two were allocated to the intervention arm and 47 allocated to control. A total of 20 participants attended at least one intervention session, and just under half of participants were followed up until 1 month post-intervention. Two female participants in London were in hospital, one male participant was in prison in North Wales, and in Glasgow, three female and three male participants were in residential rehabilitation, one male participant was in prison and one male participant was in hospital. It was not possible to conduct follow-up interviews with those participants.

Attendance for at least one intervention session was highest in London (63%) and North Wales (54%), whereas only 25% attended in Glasgow, and no participants attended in York. Follow-up at a minimum of one time point (at the end of the intervention or one month post-intervention) was also highest in London (83%) and North Wales (63%) and significantly lower in Glasgow (55%) and York (43%). Overall, men were more likely to attend at least one intervention session (44 versus 28%). Women were more likely to attend follow-up in London (85%) than in York (38%), North Wales (38%) and Glasgow (17%).

### Baseline characteristics

Overall, participants were predominantly male, in their late 30s/early 40s with a mean injecting history of between 14 and 21 years (data aggregated by gender). Baseline characteristics of the trial population by allocation and sex are presented in Table 1. Baseline characteristics were comparable between randomised treatment groups for males, despite the relatively small number of participants. The smaller group of women showed potential imbalances, e.g. a greater number of heroin users and homeless women in the intervention arm.

**Table 1:** Baseline characteristics by allocation and gender

	Male		Female	
	Intervention *N* = 34	Control *N* = 30	Intervention *N* = 18	Control *N* = 17
Age				
Mean (SD)	41.7 (6.81)	41.4 (7.30)	35.8 (6.06)	37.9 (8.79)
Median	42.5	42	35	37
Min, max	26, 57	22, 54	26, 48	26, 62
Number of years injecting				
Mean (SD)	21.4 (8.00)	19.5 (9.01)	11.9 (7.58)	16.1 (12.14)
Median	22	19	11.5	14
Min, max	3, 36	1, 42	0, 34	0, 44
Used injecting equipment provision (IEP) in the last month	31 (91.2%)	26 (86.7%)	16 (88.9%)	16 (94.1%)
Detox/maintenance drug use	26 (76.5%)	26 (86.7%)	17 (94.4%)	16 (94.1%)
Most frequently injected drug				
Heroin	15 (44.1%)	23 (76.7%)	15 (83.3%)	10 (58.8%)
Crack	1 (2.9%)	2 (6.7%)	0 (0.0%)	0 (0.0%)
Cocaine	4 (11.8%)	1 (3.3%)	0 (0.0%)	1 (5.9%)
Heroin and crack	8 (23.5%)	2 (6.7%)	3 (16.7%)	3 (17.6%)
Heroin and cocaine	1 (2.9%)	0 (0.0%)	0 (0.0%)	1 (5.9%)
Heroin and amphetamine	0 (0.0%)	0 (0.0%)	0 (0.0%)	1 (5.9%)
Speedball	1 (2.9%)	0 (0.0%)	0 (0.0%)	0 (0.0%)
Amphetamine	3 (8.8%)	2 (6.7%)	0 (0.0%)	1 (5.9%)
Methadone, M-cat	1 (2.9%)	0 (0.0%)	0 (0.0%)	0 (0.0%)
Homeless	15 (44.1%)	14 (46.7%)	8 (44.4%)	5 (29.4%)
HIV Positive	1 (2.9%)	0 (0.0%)	0 (0.0%)	0 (0.0%)
Hepatitis C Positive	17 (50%)	15 (50%)	5 (27.8%)	6 (35.3%)
Hepatitis B vaccinated	27 (79.4%)	22 (73.3%)	18 (100%)	14 (82.4%)

Compared to those who attended at least one intervention session (*n* = 20), those who did not attend any sessions (*n* = 32) were more likely to be homeless (56 vs 25%, *p* = 0.044), have injected drugs for a greater number of days in the last month (median 25 vs 6.5, *p* = 0.019) and used a greater number of needles from an IEP in the last month (median 31 vs 20, *p* = 0.056). They were more likely to be predominant heroin injectors (69 vs 40%, *p* = 0.055 for type of drug) and less likely to inject crack (31 vs 55%, *p* = 0.146) [Additional file 1]. Glasgow and York had higher levels of homelessness (68 and 52% respectively) compared to London (27%) and North Wales (29%). In addition, participants injected for a greater number of days and used more needles from an IEP [Additional file 2].

Follow-up attendance (at one or both time points) was associated with fewer days of injecting drugs in the last month (median 14 vs 27, *p* = 0.030) and fewer injections of cocaine (13 vs 30%, *p* = 0.063).

### Outcome measures

Outcome measures are summarised by randomised allocation in Table 2 (total) and Table 3 (compliance).

**Table 2:** Trial outcomes (total—groups as randomised)

	Intervention			Control		
	Baseline	End of intervention	1 month post-intervention	Baseline	End of intervention	1 month post-intervention
	*N* = 52	*N* = 24	*N* = 22	*N* = 47	*N* = 27	*N* = 23
Injecting risk practices^a^						
Mean (SD)	2.5 (2.44)	1.9 (2.16)	1.7 (2.82)	2.7 (2.93)	2.6 (2.69)	2.6 (3.20)
Median (min, max)	2 (0, 9)	1 (0, 9)	1 (0, 9)	1 (0, 9)	1 (0, 9)	1 (0, 9)
Sexual risk behaviours^b^						
Mean (SD)	3.8 (2.08)	4.3 (1.31)	4.4 (1.92)	3.8 (1.80)	3.7 (1.98)	3.1 (1.73)
Median (min, max)	5 (0, 7)	4.5 (2, 7)	5 (0, 7)	4 (0, 7)	4 (0, 6)	3 (0, 6)
Self-efficacy^c^						
Mean (SD)	24.1 (4.76)	24.8 (3.23)	25.3 (3.24)	23.9 (4.75)	23.7 (5.55)	25.0 (5.26)
Median (min, max)	25 (10, 31)	26 (17, 31)	25 (17, 32)	23 (16, 32)	23 (14, 32)	25 (11, 32)
HIV transmission knowledge^d^						
Mean (SD)	10.4 (2.53)	11.3 (1.92)	11.4 (1.59)	10.5 (2.23)	11.3 (1.98)	11.1 (2.19)
Median (min, max)	11 (4, 14)	11.5 (7, 14)	12 (7, 14)	11 (4, 14)	12 (6, 14)	12 (4, 14)
HCV transmission knowledge^e^						
Mean (SD)	23.8 (3.98)	24.9 (3.49)	24.2 (3.75)	24.8 (3.15)	25.1 (2.18)	24.3 (2.99)
Median (min, max)	24.5 (13, 30)	26 (14, 29)	24 (15, 29)	25 (20, 29)	25 (20, 29)	25 (14, 28)
HBV transmission knowledge^f^						
Mean (SD)	10.2 (3.01)	11.1 (2.10)	11.0 (2.42)	10.4 (2.45)	10.6 (2.40)	10.0 (2.70)
Median (min, max)	11 (0, 14)	11 (7, 14)	11 (7, 15)	11 (4, 14)	11 (6, 14)	11 (3, 14)
Withdrawal prevention^g^						
Mean (SD)	6.2 (4.05)	6.5 (4.19)	5.8 (3.94)	6.9 (4.32)	6.3 (4.42)	5.6 (3.45)
Median (min, max)	6 (0, 19)	6 (0, 17)	6 (0, 13)	7 (0, 17)	4 (0, 17)	5 (0, 15)
Motivation to change (for self)^h^						
Mean (SD)	4.5 (0.83)	4.5 (0.66)	4.6 (0.49)	4.4 (0.80)	4.7 (0.45)	4.6 (0.58)
Median (min, max)	5 (2, 5)	5 (3, 5)	5 (4, 5)	5 (2, 5)	5 (4, 5)	5 (3, 5)
Motivation to change (for others)^h^						
Mean (SD)	4.4 (0.82)	4.3 (0.70)	4.5 (0.51)	4.4 (0.85)	4.9 (0.36)	4.7 (0.54)
Median (min, max)	5 (2, 5)	4 (3, 5)	4.5 (4, 5)	5 (0, 5)	5 (4, 5)	5 (3, 5)

^a^Range: 0–9 (higher number = more risk events)

^b^Range: 0–7 (higher number = more risk behaviours)

^c^Range: 8–32 (higher score = greater self-efficacy)

^d^Range: 0–14 (higher score = better knowledge)

^e^Range: 0–31 (higher score = better knowledge)

^f^Range: 0–15 (higher score = better knowledge)

^g^Range: 0–20 (higher score = better prevention tactics)

^h^Range: 0–5 (higher score = more motivation)

**Table 3:** Trial outcomes (total—groups by compliance)

	Attended at least one intervention session			Attended none of the intervention sessions		
	Baseline	End of intervention	1 month post-intervention	Baseline	End of intervention	1 month post-intervention
	*N* = 20	*N* = 14	*N* = 16	*N* = 79	*N* = 37	*N* = 29
Injecting risk practices^a^						
Mean (SD)	2.3 (2.45)	1.7 (2.40)	1.4 (2.40)	2.6 (2.73)	2.5 (2.46)	2.6 (3.28)
Median (min, max)	1 (0, 9)	1 (0, 9)	1 (0, 9)	1.5 (0, 9)	1 (0, 9)	1 (0, 9)
Sexual risk behaviours^b^						
Mean (SD)	4.1 (2.04)	4.3 (1.07)	3.9 (1.89)	3.8 (1.92)	3.9 (1.90)	3.7 (1.97)
Median (min, max)	5 (0, 7)	4 (3, 6)	4 (0, 7)	4 (0, 7)	4 (0, 7)	4 (0, 7)
Self-efficacy^c^						
Mean (SD)	23.3 (5.14)	25.1 (3.12)	25.9 (3.47)	24.2 (4.64)	23.9 (5.04)	24.7 (4.76)
Median (min, max)	24.5 (12, 31)	26.5 (21, 31)	25.5 (17, 32)	24 (10, 32)	24 (14, 32)	25 (11, 32)
HIV transmission knowledge^d^						
Mean (SD)	10.8 (2.22)	11.9 (1.23)	11.4 (1.59)	10.4 (2.43)	11.1 (2.11)	11.1 (2.08)
Median (min, max)	11 (7, 14)	12 (10, 14)	12 (7, 14)	11 (4, 14)	11 (6, 14)	12 (4, 14)
HCV transmission knowledge^e^						
Mean (SD)	23.5 (3.78)	26.1 (2.53)	24.1 (3.55)	24.5 (3.59)	24.6 (2.89)	24.4 (3.06)
Median (min, max)	24 (15, 29)	26.5 (20, 29)	24 (15, 29)	25 (13, 30)	25 (14, 29)	25 (14, 29)
HBV transmission knowledge^f^						
Mean (SD)	10.3 (2.45)	11.1 (2.48)	11.1 (2.72)	10.3 (2.83)	10.7 (2.19)	10.2 (2.51)
Median (min, max)	11 (5, 13)	11.5 (7, 14)	11.5 (7, 15)	11 (0, 14)	11 (6, 14)	11 (3, 14)
Withdrawal prevention^g^						
Mean (SD)	5.4 (3.36)	6.5 (4.26)	5.9 (4.13)	6.8 (4.32)	6.4 (4.34)	5.6 (3.45)
Median (min, max)	5 (0, 12)	6.5 (1, 17)	6 (0, 13)	7 (0, 19)	6 (0, 17)	6 (0, 15)
Motivation to change (for self)^h^						
Mean (SD)	4.4 (0.82)	4.5 (0.52)	4.6 (0.51)	4.4 (0.81)	4.7 (0.58)	4.7 (0.55)
Median (min, max)	5 (2, 5)	4.5 (4, 5)	5 (4, 5)	5 (2, 5)	5 (3, 5)	5 (3, 5)
Motivation to change (for others)^h^						
Mean (SD)	4.4 (0.59)	4.2 (0.70)	4.4 (0.51)	4.4 (0.88)	4.8 (0.49)	4.7 (0.53)
Median (min, max)	4 (3, 5)	4 (3, 5)	4 (4, 5)	5 (1, 5)	5 (3, 5)	5 (3, 5)

^a^Range: 0–9 (higher number = more risk events)

^b^Range: 0–7 (higher number = more risk behaviours)

^c^Range: 8–32 (higher score = greater self-efficacy)

^d^Range: 0–14 (higher score = better knowledge)

^e^Range: 0–31 (higher score = better knowledge)

^f^Range: 0–15 (higher score = better knowledge)

^g^Range: 0–20 (higher score = better prevention tactics)

^h^Range: 0–5 (higher score = more motivation)

The summary of group differences based on the exploratory longitudinal regression analyses for each outcome (Table 4) revealed improved (fewer) injecting risk practices, improved self-efficacy, better hepatitis C and hepatitis B transmission knowledge and greater use of withdrawal prevention techniques in the intervention arm. Little change for any group was seen for HIV transmission knowledge. A number of results appeared counterintuitive. Participants in the randomised intervention group engaged in a greater number of sexual risk behaviours at both follow-up time points, although group differences were reduced to minimal in the attendance-based analysis. Motivation to change was highly skewed, with most participants indicating being highly motivated.

**Table 4:** Summary of mean group differences for outcome measures^i^

		Analysis by randomised groups (ITT)			Analysis by attendance of at least one intervention session		
		Mean	95% CI	80% CI	Mean	95% CI	80% CI
Injecting risk practices^a^	End of intervention	−0.45	−1.50 to 0.61	−1.14 to 0.24	−0.52	−1.78 to 0.74	−1.35 to 0.30
	1 month post-intervention	−0.25	−1.33 to 0.82	−0.96 to 0.45	−0.25	−1.51 to 1.01	−1.08 to 0.57
Sexual risk behaviours^b^	End of intervention	0.57	−0.20 to 1.34	0.06 to 1.07	0.08	−0.85 to 1.02	−0.53 to 0.70
	1 month post-intervention	1.26	0.43 to 2.08	0.71 to 1.80	0.13	−0.80 to 1.06	−0.48 to 0.74
Self-efficacy^c^	End of intervention	1.17	−0.71 to 3.05	−0.06 to 2.40	2.20	0.02 to 4.38	0.77 to 3.62
	1 month post-intervention	0.08	−1.90 to 2.07	−1.22 to 1.38	1.65	−0.51 to 3.82	0.24 to 3.07
HIV transmission knowledge^d^	End of intervention	−0.06	−0.88 to 0.75	−0.60 to 0.47	0.04	−0.91 to 0.99	−0.58 to 0.66
	1 month post-intervention	0.18	−0.70 to 1.06	−0.39 to 0.76	−0.07	−1.00 to 0.87	−0.68 to 0.55
HCV transmission knowledge^e^	End of intervention	0.16	−1.37 to 1.68	−0.84 to 1.15	2.13	0.41 to 3.85	1.01 to 3.26
	1 month post-intervention	0.12	−1.52 to 1.75	−0.96 to 1.19	0.30	−1.40 to 1.99	−0.81 to 1.41
HBV transmission knowledge^f^	End of intervention	0.79	−0.31 to 1.89	0.07 to 1.51	0.79	−0.51 to 2.08	−0.06 to 1.63
	1 month post-intervention	0.75	−0.41 to 1.91	−0.01 to 1.51	0.88	−0.41 to 2.18	0.03 to 1.73
Withdrawal prevention^g^	End of intervention	0.28	−1.37 to 1.93	−0.80 to 1.36	0.38	−1.54 to 2.31	−0.88 to 1.64
	1 month post-intervention	1.41	−0.34 to 3.17	0.26 to 2.57	1.83	−0.10 to 3.76	0.57 to 3.09
Motivation to change (for self)^h^	End of Intervention	−0.20	−0.47 to 0.07	−0.38 to −0.03	−0.21	−0.52 to 0.09	−0.42 to −0.01
	1 month post-intervention	−0.01	−0.30 to 0.28	−0.20 to 0.18	−0.21	−0.51 to 0.10	−0.41 to −0.01
Motivation to change (for others)^h^	End of intervention	−0.40	−0.67 to-0.13	−0.58 to −0.22	−0.53	−0.84 to −0.23	−0.73 to −0.33
	1 month post-intervention	−0.14	−0.43 to 0.15	−0.33 to 0.05	−0.29	−0.59 to 0.01	−0.49 to −0.10

^a^Range: 0–9 (higher number = more risk events)

^b^Range: 0–7 (higher number = more risk behaviours)

^c^Range: 8–32 (higher score = greater self-efficacy)

^d^Range: 0–14 (higher score = better knowledge)

^e^Range: 0–31 (higher score = better knowledge)

^f^Range: 0–15 (higher score = better knowledge)

^g^Range: 0–20 (higher score = better prevention tactics)

^h^Range: 0–5 (higher score = more motivation)

^i^Mean differences represent the estimated mean group difference following regression analysis adjusted for outcome at baseline, gender and recruitment site; Positive mean difference = higher score in the intervention arm, negative mean difference = higher score in the control arm

Sample sizes were too small to investigate possible interactions with baseline characteristics and outcomes, e.g. whether score changes can only be seen in a subset of the participant population.

All outcome measures were reviewed with regard to the number of missing items that contribute to each outcome. Overall, data completeness was very high across all questionnaires responses, and most items were only missing sporadically.

At 1 month post-intervention, no increase in self-reported injecting in more “risky” sites (e.g. groin, neck) was observed among participants who had attended at least one session of the intervention. A trend towards injecting on fewer days in the past 28 days for those who had attended at least one session at 1 month post-intervention was seen. Therefore, exposure to sessions on improving injecting techniques as part of BBV harm reduction psychosocial intervention does not appear to encourage riskier injecting practices or frequency of injecting.

No adverse events were recorded as a result of participating in the feasibility trial.

### Health economics

#### Service use questionnaire

Analysis of the questionnaires identified several categories that could be excluded from the assessment battery in a full-randomised controlled trial. Twelve service use categories of cost were identified where >90% of responses at all three contacts were zero. The results allow questionnaires to be revised for future use.

Costs for sessions 1, 2 and 3 are estimated for each of the treatment centres (Table 5). Cost per patient is attributed to the attendee and then the cost per session summed to derive a total treatment cost. Total patient treatment costs are derived by summing the costs of the sessions attended (maximum = 3).

**Table 5:** Intervention and control costs per session by centre

Intervention session costs				
	Total cost	Patients attending	Cost per patient	Cost excl. training
London				
Session 1	£349.02	6	£58.17	£30.21
Session 2	£333.35	5	£66.67	£33.12
Session 3	£333.20	5	£66.64	£33.09
London (2)				
Session 1	£316.56	2	£158.28	£74.39
Session 2	£323.43	3	£107.81	£51.89
Session 3	£316.44	2	£158.22	£74.33
Scotland				
Session 1	£310.83	3	£103.61	£47.69
Session 2	£310.38	3	£103.46	£47.54
Session 3	£308.80	2	£154.40	£70.51
Wales				
Session 1	£318.48	6	£53.08	£25.11
Session 2	£313.16	4	£78.29	£36.55
Session 3	£319.38	6	£53.23	£53.23
Control cost				
Cost item			Unit cost	
Staff time			£0.81	
Leaflet			£0.05	
Cost per patient			£0.86	

Mean cost was £58.17 for patients attending one session, £148.54 for those attending two sessions and £270.67 for those attending all three sessions in the intervention group. Control cost per patient was £0.86. In a pragmatic setting, these sessions would be delivered to more patients, thus reducing the mean per session training cost.

#### Health-related quality of life

EQ-5D-5 L scores were valued using the social tariff [36] at the three time points using paired analysis (Table 6). The tariff provides utility values from a population survey whereby values for each health state are given a utility score; hence, these scores reflect the population preferences for health state values.

**Table 6:** EQ-5D-5 L tariff scores at baseline and follow-up

	EQ-5D-5 L tariff score (s.d.)		
	Baseline	End of intervention	1 month post-intervention
Control	0.617 (0.323) *N* = 47	0.646 (0.314) *N* = 47	0.788 (0.258) *N* = 47
Intervention	0.672 (0.247) *N* = 52	0.754 (0.193) *N* = 52	0.775 (0.256) *N* = 52
	EQ-5D-5 L changes		
	Baseline to End of intervention	End of intervention to 1 month post-intervention	
Control	+0.0738 (0.216) *N* = 26	+0.1420 (0.375) *N* = 17	
Intervention	+0.0273 (0.233) *N* = 24	+0.0369 (0.232) *N* = 17	

Baseline and control showed increases in scores on EQ-5D-5L across the time period. EQ-5D-5L scores in both groups improved from baseline through the two follow-ups showing potential for health improvement and associated QALY gains. Differences between groups should be treated with caution due to the small sample size. Differences in the changes between groups were not significant, for the change baseline to the end of the intervention the mean difference between groups was 0.05 (95% CI: −0.08, 0.17) and from end of the intervention to 1 month post-intervention the difference between groups was 0.11 (95% CI: −0.11, 0.32). We do not present quality-adjusted life years due to the short follow-up and the expectation that health utility gains would become evident over a period longer than 1 month.

#### Health and social resources used

Although wider NHS costs, social costs and criminal justice costs also showed a reduction from baseline through follow-up periods, there were no significant differences between groups at any time point (Table 7). Health care and criminal justice costs were also assessed at baseline and follow-up by compliance, but there were no significant differences based on whether a patient had attended one or more treatment sessions compared to those who had attended no sessions.

**Table 7:** Wider health care, criminal justice and societal costs (2014/5 prices) mean cost (s.d.) per patient

	Baseline		End of intervention		1 month post-intervention	
	Intervention	Control	Intervention	Control	Intervention	Control
Total wider health care Cost	£1109 (1696.14)	£1257 (2177.61)	£705 (673.39)	£997 (786.04)	£662 (682.47)	£1466 (2885.66)
Difference between groups	£148 95% CI: (−657.94, 954.54)		£292 95% CI: (−137.81, 721.34)		£804 95% CI: (−611.92, 2220.61)	
Total criminal justice cost	£1239 (2581.51)	£1284 (3953.47)	£439 (2060.80)	£289 (1348.13)	£236 (1053.86)	£521 (1465.97)
Difference between groups	£45 95% CI: (−1344.95, 1434.14)		−£151 95% CI: (−1191.45, 890.34)		£285 95% CI: (−520.06, 1091.05)	
Total social cost	£2489 (3397.65)	£2494 (4498.24)	£1194 (2178.38)	£1328 (1563.11)	£908 (1279.79)	£1909 (3077.46)
Difference between groups	£5 95% CI: (−2107.85, 2117.58)		£134 95% CI: (−1034.59, 1303.28)		£1001 95% CI: (−662.53, 2665.44)	

### Acceptability of the PROTECT intervention

Intervention group participants who attended the PROTECT sessions rated the sessions highly, reporting they had gained valuable knowledge on blood-borne virus transmission, safer drug use, hygiene and handwashing, cleaning equipment and preparing for risk situations such as withdrawal. To improve the PROTECT intervention, participants suggested making it more visual, interactive and incorporating more practical instruction around injecting technique and injecting sites. It was also suggested that the videos illustrating the side effects of injecting should be more graphic and feature real people rather than animations.

Facilitators who delivered the PROTECT intervention suggested delivering the training event over 2 days, with equal time devoted to each of the three PROTECT sessions, incorporating opportunities for mock delivery. They appreciated peer educators being involved in the training event and that their input had been incorporated into the final version of the PROTECT manual. The sessions were rated highly and being involved in the intervention had improved knowledge and led to changes in their practice with clients from IEP. Session 1 was thought too lengthy and facilitators were less comfortable delivering the didactic parts and discussing sexual risk behaviour. Making the intervention more interactive and including specialist workers for specific components (e.g. injecting instructors, BBV nurses or sexual health practitioners) were suggested improvements. Other potential modes of delivery were delivery in bite-size pieces to clients, developing as an app or QR scanner, or as an online resource for staff training. Preparedness plans could also be incorporated into clients’ care plans. Identified key target groups were new referrals to treatment, new injectors, sex workers, people who inject who engage in chemsex, ie. the use of drugs (most commonly crystal methamphetamine, mephedrone and gammaydroxybutrate/gamma-butyrolactone), by men who have sex with men to facilitate or enhance sexual activity.

## Discussion

We explored the feasibility of a three-session, gender-specific psychosocial group intervention to reduce BBV transmission behaviours among PWID which included skills to improve injecting techniques and thus vein care, and strategies to avoid and plan for risk situations that PWID had themselves identified within in-depth interviews undertaken to inform the intervention development (see the “Methods” section).

Although the resultant intervention was acceptable to both facilitators and attending participants and 57% of eligible participants agreed to be randomised, suggesting support for addressing BBV risk behaviours among PWID, there were considerable difficulties recruiting particular groups of PWID, mainly women and new injectors. One potential way to improve recruitment could have been to use chain or snowball sampling, rather than researcher recruitment, where recruited participants are encouraged to recruit members of their networks to the study. A previous survey in Wales suggested individuals whose main source of needles and syringes was secondary distribution were more likely to be younger and more recent onset injectors; this might explain the difficulty in recruiting newer and younger injectors [37]. Research suggests that women are more likely than men to face additional barriers to accessing and attending treatment for drug use including family and childcare responsibilities, shame or fear that their children will be removed [38, 39]. Observations from researchers suggest that male partners often accompanied women to the harm reduction services (including prevention, treatment and IEP). The prevalence of intimate partner violence victimisation among female drug users is high [16]; therefore, it is possible that in some cases, male partners prevented women from entering the study. Women-only treatment programmes are recommended and may show improved drug use outcomes [40]; however, interventions and services need to be cognisant of the potential role of intimate partners in accessing treatment.

The proportion attending at least one session in our study was low with just 38% overall attending at least one session (44% of males and 28% of females). While our adherence rates are lower than previous trials of behavioural group interventions to address BBV among PWID (range 56–86%) [41–44], these trials recruited participants entering or engaged in drug treatment which may account for the difference. All of our participants had injected drugs within the past 30 days and 44% were homeless. Similar to other trials, we found that PWID who were homeless or who injected more frequently were less likely to participate or be followed up [42, 45]. Despite gender-specific sessions being offered [46], women were less likely to attend at least one intervention session than men in our study. Potential reasons for this were previously discussed.

Although the findings suggest that the PROTECT intervention has the potential to positively influence some PWID BBV risk behaviour, non-attendance at the intervention at the York site substantially influenced the results, highlighting the need for flexible delivery of the intervention content to ensure wider reach. Intervention delivery proved more feasible in London than the other sites, with high attendance at the intervention and higher follow-up rates. Participants from Glasgow and York reported higher levels of homelessness, and participants had injected for a greater number of days and used more needles from an IEP, which may have contributed towards lower attendance rates. In addition, text message reminders were sent about session times and dates from the service (reported preference of participants) at the York site; whereas in the other sites, the researcher contacted participants by telephone to remind them a day in advance plus a reminder text on the day. Moreover, staff from the local Clinical Research Network were responsible for recruitment and follow-up of participants (due to researcher leaving); whereas in other sites, participants had contact with the same named researcher throughout, with this established relationship possibly contributing to increased attendance. In addition, reimbursement for travel costs (bus tickets), time and contingency management were paid in cash in the London site versus high street vouchers at the other three sites and peer-educators co-facilitated the intervention in the London site only.

Overall, recruitment and retention rates achieved in this feasibility trial lead us to conclude that progression to a full trial is not recommended. There are many factors that may have contributed to the different uptake and retention across sites, and therefore, it is not possible to provide a definitive explanation of the differences in rates reported. However, it appears that the complex needs of many PWID may have limited engagement of those potentially most at risk of engaging in BBV transmission behaviours (e.g. homeless PWID, more frequent injectors, crack use).

The importance of management and of service staff buy-in was stressed by the researchers; presenting the study at staff meetings was used in some settings. In addition, facilitators valued being involved in the development of the intervention. Training of intervention facilitators should be delivered locally (we carried this out centrally in London creating challenges for more distantly located staff) and we recommend that sufficient time be allocated to allow quality assurance of the delivery of the intervention, before the intervention is delivered in practice. Identifying sites that have previously been involved in similar research may facilitate trial implementation as the service will be familiar with what involvement in research studies and trials entails.

The content of the intervention was rated highly by facilitators and intervention participants alike and there was support for addressing symbiotic goals, planning for risk behaviours and teaching injecting skills to PWID [47–49]. Indeed some intervention participants stressed the need for more practical assistance on injecting technique, including observation and feedback on their own injecting technique. Facilitators felt that the manual could be improved by being more flexible, allowing facilitators to cover the information in each section without having to follow the text verbatim. Both facilitators and participants felt the intervention could be more visual and interactive. Making it available online and including information on novel psychoactive substances was considered a way of making the intervention more relevant and attractive to younger people.

For those participants who attended the intervention sessions, all candidate outcome measures had very good completion rates. The number of injecting risk practices, and self-efficacy in particular, showed improvements in the intervention group that were maintained up to 1 month follow-up. These outcomes might be considered in a larger scale study in the future. BBV transmission knowledge was more likely to show short term improvements only, whereas withdrawal prevention questions had only limited applicability in this study population.

A meta-analysis found that the incidence of HCV reinfection following successful treatment for HCV among PWID was 2.4/100 per year, and 6.4/100 per year among those who reported injecting drug use post sustained viral response (SVR) [8]. Although there is low risk of reinfection following successful treatment for HCV, a large, cohort study conducted in Scotland found that despite achieving the optimal treatment outcome, a significant minority of PWID continued to inject post-SVR at an intensity which lead to either hospitalisation or death and increased risk of reinfection [35]. These findings highlight that “harm reduction interventions aimed at reducing the risk of HCV transmission should also continue to be promoted once treatment ceases” [50].

## Conclusions

While the intervention showed the potential to positively influence BBV risk behaviours, the findings demonstrate that a future definitive RCT of the PROTECT intervention is not currently feasible in the UK. Despite this, considerable and valuable insight has been obtained showing the need for a greater embedding of BBV risk reduction in the work of substance misuse services and highlights an urgent unmet health need for PWID. Furthermore, the research provides a body of evidence as to how this might best be achieved, and has generated important learning about the feasibility, delivery and implementation of the PROTECT intervention which should inform future studies in the field.

Future studies could consider the use of “chain referral sampling” where existing study participants recruit future participants from among their acquaintances to target participants who may be hidden or difficult to reach for researchers. Participants who assist with the recruitment of other participants would be rewarded for every additional participant they helped recruit. All the participating harm reduction services suggested there was benefit in refining the intervention further by adapting it for delivery in specific settings (e.g. IEP, pharmacy IEP, prison) and to specific groups of PWID including those living in homeless hostels, people receiving opiate substitution therapy, young injectors when they are transferred from adolescent to adult addiction services, steroid injectors, those engaged in chemsex and those injecting novel psychoactive substances.

## Additional files

Additional file 1: Participant characteristics by compliance (Total). (DOCX 14 kb)
Additional file 2: Participant characteristics by recruitment Site. (DOCX 15 kb)

## Abbreviations

BBV: Blood-borne viruses
EQ-5D-5L: European quality of life-5 dimensions-5 levels version
HBV: Hepatitis B virus
HCV: Hepatitis C virus
HIV: Human immunodeficiency virus
HRQoL: Health-related quality of life
IEP: Injecting equipment provision
PWID: People who inject drugs
QALY: Quality adjusted life year
TAU: Treatment as usual
