# Supplementary material for: The acceptability and feasibility of a brief psychosocial intervention to reduce blood-borne virus risk behaviours among people who inject drugs: a randomised control feasibility trial of a psychosocial intervention (the PROTECT study) versus treatment as usual
Source: Harm Reduct J. 2017 Mar 21;14:14. doi: 10.1186/s12954-017-0142-5 (PMC5359828; doi:10.1186/s12954-017-0142-5)
Supplement: Additional file 1: — Participant characteristics by compliance (Total). (DOCX 14 kb) [file 12954_2017_142_MOESM1_ESM.docx]

Additional file 1: Participant Characteristics by Compliance (Total)

|  | **Attended at least one intervention session**  **N=20** | **Attended no intervention sessions**  **N=32** |
| --- | --- | --- |
| Gender |  |  |
| Male | 15 (75%) | 19 (59%) |
| Female | 5 (25%) | 13 (41%) |
| Transgender | 0 (0%) | 0 (0%) |
| Age |  |  |
| Mean (SD) | 41.5 (7.29) | 38.5 (6.85) |
| Median | 41.5 | 39 |
| Min, Max | 29, 57 | 26, 50 |
| Number of years since first injected |  |  |
| Mean (SD) | 20.1 (8.12) | 16.9 (9.45) |
| Median | 22 | 18.5 |
| Min, Max | 8, 36 | 0, 34 |
| Homeless | 5 (25%) | 18 (56%) |
| Number of days injected drugs in last month |  |  |
| Mean (SD) | 13.0 (11.00) | 19.3 (9.49) |
| Median | 6.5 | 25 |
| Min, Max | 1, 28 | 3, 28 |
| Most frequently injected drug |  |  |
| Heroin | 8 (40%) | 22 (69%) |
| Crack | 1 (5%) | 0 (0%) |
| Cocaine | 2 (10%) | 2 (6%) |
| Heroin & Crack | 8 (40%) | 3 (9%) |
| Heroin & Cocaine | 0 (0%) | 1 (3%) |
| Speedball | 0 (0%) | 1 (3%) |
| Amphetamine | 1 (5%) | 2 (6%) |
| Methadone, M-cat | 0 (0%) | 1 (3%) |
| Any drugs injected in the last month |  |  |
| Heroin | 18 (90%) | 30 (94%) |
| Crack | 11 (55%) | 10 (31%) |
| Amphetamine (Speed) | 1 (5%) | 3 (9%) |
| Ketamine | 1 (5%) | 0 (0%) |
| Methadone | 0 (0%) | 0 (0%) |
| Cocaine | 4 (20%) | 8 (25%) |
| Mephedrone (m-cat) | 0 (0%) | 1 (3%) |
| Methamphetamine | 0 (0%) | 0 (0%) |
| Other | 1 (5%) | 1 (3%) |
| Injected heroin and cocaine in last month (Speedball) | 9 (45%) | 12 (38%) |
| Any drugs used in the last month |  |  |
| Cocaine | 11 (55%) | 12 (38%) |
| Amphetamine | 2 (10%) | 4 (13%) |
| Crack | 16 (80%) | 23 (72%) |
| Heroin | 16 (80%) | 31 (97%) |
| Mephedrone (m-cat) | 1 (5%) | 2 (6%) |
| Methamphetamine | 0 (0%) | 0 (0%) |
| Ecstasy / E | 2 (10%) | 2 (6%) |
| Cannabis | 13 (65%) | 17 (53%) |
| Solvents or Glue | 0 (0%) | 0 (0%) |
| Ketamine | 1 (5%) | 0 (0%) |
| Benzodiazepines | 11 (55%) | 18 (56%) |
| Other drugs | 1 (5%) | 7 (22%) |
| Used injecting equipment provision (IEP) in the last month | 17 (85%) | 30 (94%) |
| Number of individual needles |  |  |
| Mean (SD) | 31.3 (30.60) | 61.5 (70.86) |
| Median | 20 | 31 |
| Min, Max | 10, 100 | 8, 280 |
| Current detox/maintenance drug use | 16 (80%) | 27 (84%) |
| Length of time on current script |  |  |
| Less than a month | 1 (5%) | 1 (3%) |
| 1 to 6 months | 2 (10%) | 8 (25%) |
| Over 6 months | 14 (70%) | 18 (56%) |
| HIV Positive | 1 (5%) | 0 (0%) |
| Hepatitis C Positive | 7 (35%) | 15 (47%) |
