# Supplementary material for: The acceptability and feasibility of a brief psychosocial intervention to reduce blood-borne virus risk behaviours among people who inject drugs: a randomised control feasibility trial of a psychosocial intervention (the PROTECT study) versus treatment as usual
Source: Harm Reduct J. 2017 Mar 21;14:14. doi: 10.1186/s12954-017-0142-5 (PMC5359828; doi:10.1186/s12954-017-0142-5)
Supplement: Additional file 2: — Participant characteristics by recruitment Site. (DOCX 15 kb) [file 12954_2017_142_MOESM2_ESM.docx]

Additional file 2: Participant Characteristics by Recruitment Site

|  | **London**  **N=30** | **York**  **N=23** | **Glasgow**  **N=22** | **North Wales**  **N=24** |
| --- | --- | --- | --- | --- |
| Gender |  |  |  |  |
| Male | 17 (57%) | 15 (65%) | 15 (68%) | 16 (67%) |
| Female | 13 (43%) | 8 (35%) | 6 (27%) | 8 (33%) |
| Transgender | 0 (0%) | 0 (0%) | 1 (5%) | 0 (0%) |
| Age |  |  |  |  |
| Mean (SD) | 42.8 (7.53) | 37.6 (6.13) | 38.3 (6.85) | 39.8 (8.33) |
| Median | 42 | 37 | 40 | 42 |
| Min, Max | 29, 62 | 27, 50 | 26, 50 | 22, 56 |
| Number of years since first injected |  |  |  |  |
| Mean (SD) | 20.6 (11.27) | 15.9 (9.05) | 17.6 (8.97) | 17.9 (8.10) |
| Median | 22 | 17 | 16 | 18.5 |
| Min, Max | 0, 40 | 0, 32 | 4, 35 | 1, 32 |
| Homeless | 8 (27%) | 12 (52%) | 15 (68%) | 7 (29%) |
| Number of days injected drugs in last month |  |  |  |  |
| Mean (SD) | 18.1 (9.87) | 19.0 (10.28) | 22.1 (9.48) | 10.3 (8.83) |
| Median | 20 | 26 | 28 | 8 |
| Min, Max | 2, 28 | 2, 28 | 1, 28 | 1, 28 |
| Most frequently injected drug |  |  |  |  |
| Heroin | 15 (50%) | 16 (70%) | 16 (73%) | 16 (67%) |
| Crack | 2 (7%) | 0 (0%) | 0 (0%) | 1 (4%) |
| Cocaine | 0 (0%) | 0 (0%) | 6 (27%) | 0 (0%) |
| Heroin & Crack | 11 (37%) | 3 (13%) | 0 (0%) | 2 (8%) |
| Heroin & Cocaine | 2 (7%) | 0 (0%) | 0 (0%) | 0 (%) |
| Heroin & Amphetamine | 0 (0%) | 0 (0%) | 0 (0%) | 1 (4%) |
| Speedball | 0 (0%) | 1 (4%) | 0 (0%) | 0 (0%) |
| Amphetamine | 0 (0%) | 3 (13%) | 0 (0%) | 3 (13%) |
| Methadone, M-cat | 0 (0%) | 0 (0%) | 0 (0%) | 1 (4%) |
| Any drugs injected in the last month |  |  |  |  |
| Heroin | 30 (100%) | 22 (96%) | 20 (91%) | 20 (83%) |
| Crack | 18 (60%) | 12 (52%) | 2 (9%) | 10 (42%) |
| Amphetamine (Speed) | 0 (0%) | 7 (30%) | 1 (5%) | 7 (29%) |
| Ketamine | 1 (3%) | 0 (0%) | 0 (0%) | 1 (4%) |
| Methadone | 0 (0%) | 0 (0%) | 0 (0%) | 0 (0%) |
| Cocaine | 2 (7%) | 2 (9%) | 12 (55%) | 3 (13%) |
| Mephedrone (m-cat) | 0 (0%) | 0 (0%) | 0 (0%) | 1 (4%) |
| Methamphetamine | 1 (3%) | 0 (0%) | 1 (5%) | 0 (0%) |
| Other | 3 (10%) | 1 (4%) | 1 (5)% | 0 (0%) |
| Injected heroin and cocaine in last month (Speedball) | 15 (50%) | 11 (48%) | 5 (23%) | 9 (38%) |
| Any drugs used in the last month |  |  |  |  |
| Cocaine | 15 (50%) | 5 (22%) | 13 (59%) | 9 (38%) |
| Amphetamine | 1 (3%) | 7 (30%) | 1 (5%) | 10 (42%) |
| Crack | 29 (97%) | 14 (61%) | 12 (55%) | 19 (79%) |
| Heroin | 29 (97%) | 22 (96%) | 21 (95%) | 19 (79%) |
| Mephedrone (m-cat) | 2 (7%) | 0 (0%) | 3 (14%) | 5 (21%) |
| Methamphetamine | 1 (3%) | 1 (4%) | 2 (9%) | 0 (0%) |
| Ecstasy / E | 2 (7%) | 0 (0%) | 4 (18%) | 1 (4%) |
| Cannabis | 23 (77%) | 12 (52%) | 18 (82%) | 8 (33%) |
| Solvents or Glue | 0 (0%) | 0 (0%) | 0 (0%) | 0 (0%) |
| Ketamine | 2 (7%) | 0 (0%) | 0 (0%) | 0 (0%) |
| Benzodiazepines | 12 (40%) | 10 (43%) | 20 (91%) | 17 (71%) |
| Other drugs | 5 (17%) | 6 (26%) | 5 (23%) | 3 (13%) |
| Used injecting equipment provision (IEP) in the last month | 28 (93%) | 21 (91%) | 22 (100%) | 18 (75%) |
| Number of individual needles |  |  |  |  |
| Mean (SD) | 51.3 (50.43) | 49.7 (48.73) | 96.1 (176.05) | 41.7 (50.11) |
| Median | 34 | 40 | 48 | 28 |
| Min, Max | 0, 220 | 8, 210 | 1, 840 | 0, 200 |
| Current detox/maintenance drug use | 29 (97%) | 22 (96%) | 19 (86%) | 15 (63%) |
| Length of time on current script |  |  |  |  |
| Less than a month | 2 (7%) | 3 (13%) | 0 (0%) | 2 (8%) |
| 1 to 6 months | 2 (7%) | 7 (30%) | 5 (23%) | 2 (8%) |
| Over 6 months | 25 (83%) | 12 (52%) | 14 (64%) | 12 (50%) |
| HIV Positive | 0 (0%) | 0 (0%) | 1 (5%) | 0 (0%) |
| Hepatitis C Positive | 12 (40%) | 10 (43%) | 16 (73%) | 5 (21%) |
